# Can Intersectoral Interventions Reduce Substance Use in Adolescence? Evidence From a Multicentre Randomized Controlled Study

**DOI:** 10.3389/ijph.2022.1604677

**Published:** 2022-08-26

**Authors:** Sara Valente de Almeida, Rafael Correa, Judite Gonçalves

**Affiliations:** ^1^ School of Public Health, Imperial College London, London, United Kingdom; ^2^ Nova School of Business and Economics, Universidade Nova de Lisboa, Lisbon, Portugal; ^3^ Escola de Enfermagem de Ribeirão Preto, Universidade de São Paulo, São Paulo, Brazil; ^4^ Centro Universitário Assis Gurgacz, Cascavel, Brazil; ^5^ Faculdade de Motricidade Humana, Universidade de Lisboa, Lisbon, Portugal

**Keywords:** LMICs, adolescent health, substance use, adolescent behavior, RCT, interdisciplinary science

## Abstract

**Objectives:** We measure the impacts of an intersectoral intervention tackling adolescent substance use implemented between 2017 and 2019 in a tri-border region of Brazil, Paraguay, and Argentina.

**Methods:** The intervention involved 23 institutions from different sectors and 880 adolescents, equally split between randomly selected treatment and control classes across institutions. Treatment group students were involved in the co-development of activities to tackle substance use within their institutions. Both treatment and control group students benefited from the activities developed and implemented from the second year of the intervention. We use difference-in-differences models to measure the impacts of participation in the co-development of the activities on alcohol, tobacco, and cannabis consumption.

**Results:** Adolescents involved in the co-development of activities are 8 pp less likely to consume tobacco and cannabis, and 13 pp less likely to consume alcohol (p < 0.01), compared to those who only participate in the activities. Among cannabis users, frequent consumption is also reduced by the intervention. Peer frequency of consumption is strongly associated with individual consumption.

**Conclusion:** Co-development of activities by the subjects themselves can be key to decreasing substance use in this very crucial stage of life, especially if the institutions and the implementers are familiar with the area and subjects of the intervention.

## Introduction

Adolescence is a stage of life when individuals are more likely to engage in risky behavior, and when substance use tends to increase [1]. Tobacco, alcohol, and cannabis consumption among teenagers can have devastating health consequences and jeopardize users’ professional and personal prospects [2–5]. Policies aiming to discourage initiation and decrease substance use should focus on this specific age group, where intervention is likely to be more effective [6, 7]. Moreover, tackling substance use at this critical developmental stage is key for empowering and providing youths with opportunities to grow healthy and successful in both professional and personal aspects of life. This study assesses the effectiveness of an intervention tackling alcohol, tobacco, and cannabis consumption among teenagers in a tri-border region of Brazil, Argentina and Paraguay.

Engagement in consumption of tobacco, alcohol, and cannabis is associated with a series of social factors. Migration, in particular, can be a catalyst for consumption, partly due to lack of parental control [8, 9]. Peer influence and the social environment are also key determinants of substance use [10]. The present study analyses an intervention that took place in neighbouring *Foz do Iguaçú*, Brazil, Puerto Iguazu in Argentina, and Caaguazu in Paraguay. Due to its geographical characteristics and weak local governance, this region constitutes a “perfect storm” of critical factors like criminal activity and socioeconomic disadvantage [11]. In this complex and rather unique environment, the risk factors for early substance use are abundant.

Targeting vulnerable groups living in complex environments should be a priority, to create sustainable investments in adolescent health at local, national, and global levels [12–14]. Governments and social actors must adopt a holistic approach to address the triggers of substance use and provide adequate assistance to teenagers, to prevent initiation and reduce substance consumption. This can be done through intersectoral actions, i.e., actions targeting health outcomes undertaken by sectors other than the health sector, like the education sector [15]. Available reviews of interventions to prevent substance (ab)use among adolescents suggest that school-based interventions are effective [6, 7, 13], especially programs that are highly interactive, skills-focused, and implemented over multiple years [7]. Yet, most existing evidence on substance abuse interventions comes from high-income countries, there is lack of evidence on differential effects of interventions by gender or socioeconomic status, and little is known about the effectiveness of specific intervention components [6]. Overall, especially in low-income regions, there is a need to implement different types of (school-based) interventions to learn what kind of activities work, at what ages, and how they help adolescents of different sociodemographic groups.

In this study, we measure the effectiveness of a randomized controlled intersectoral intervention tackling alcohol, tobacco, and cannabis consumption among teenagers in a low-income, complex region of Brazil, Argentina and Paraguay. The intervention took place over 3 years and involved students from different schools and social organizations in the co-development of a set of activities, designed to raise awareness about substance use and keep students occupied after classes. We evaluate specifically the effectiveness of student co-development of the activities. In addition, we identify the main sociodemographic characteristics associated with substance use.

Main results show that the intervention (i.e., involvement in the co-development of activities) reduced the likelihood of any alcohol, tobacco, and cannabis consumption, as well as the likelihood of frequent cannabis use among users. Peer effects (i.e., average frequency of consumption among peers) were statistically associated with higher likelihood of (more frequent) use.

This study is organized as follows: Section 2 presents the study design and statistical methods, Section 3 contains the main results, and Section 4 discusses the results and concludes.

## Methods

### Study Design

This study evaluates a multicentre randomized controlled experiment conducted between 2017 and 2019. In total, 880 students from Brazil, Paraguay, and Argentina participated, with around 67% participating in all three data collection waves (see below). The study was approved by the research ethics committee of the State University of the West of Paraná (CAAE 82847418.6.0000.0107) and registered according to CONSORT guidelines (UTN U1111=1252-6877) [16].

The research team identified 23 institutions and 115 active individuals (i.e., social workers, educators, and other collaborators that worked closely with the institutions), which were selected to be the “project implementers.” The institutions that partnered with this project are dedicated to providing teenagers from local communities with social support at different levels, e.g., sports centres and institutions focused on inclusion and substance abuse. All institutions’ representatives signed a participation form and provided a list of potential project implementers. The eligibility criterion for being an implementer was being a member of the institution, and their role was to support the development of activities with adolescents (as well as responsibility for data collection).

In each institution, classes were set and randomized into treatment and control classes, with a total of 440 adolescents allocated to treatment and 440 to control classes. The final sample was composed of 880 adolescents from 14 to 17 years old: 376 Brazilians (42.7%), 292 Paraguayans (24.1%), and 212 Argentinians (33.2%) (for more details see Supplementary Table S1).

The intervention took place over the course of 3 years, starting in 2017. The structure of the intervention was the same in each year and consisted in three meetings of 120 min focused on the following themes: 1) vulnerability and health care network, 2) analysis of adolescent health-related indicators and 3) strategic planning and development of proposals for future activities. These three meetings happened during the first quarter of the year with 1 month interval between them.

More specifically, the first meeting between the project implementers and the adolescents in the treatment group worked as an “idea incubator,” where teams discussed the development and implementation of new extra curricular activities, to be offered at their institution during the following year. After that, and before the second meeting all students (treatment and control) answered an electronic survey about their substance consumption, mental health, physical activity, and relationship with their parents. The second meeting, consisted in having students in the treatment group analyse the results from the electronic survey (for all students). This analysis then informed the development of the activities, by identifying the main priorities by institution and action area (Supplementary Table S2) [17]. Lastly, the third meeting was dedicated to writing concrete proposals for the new extra curricular activities to be delivered from the following year.

**FIGURE 1:**
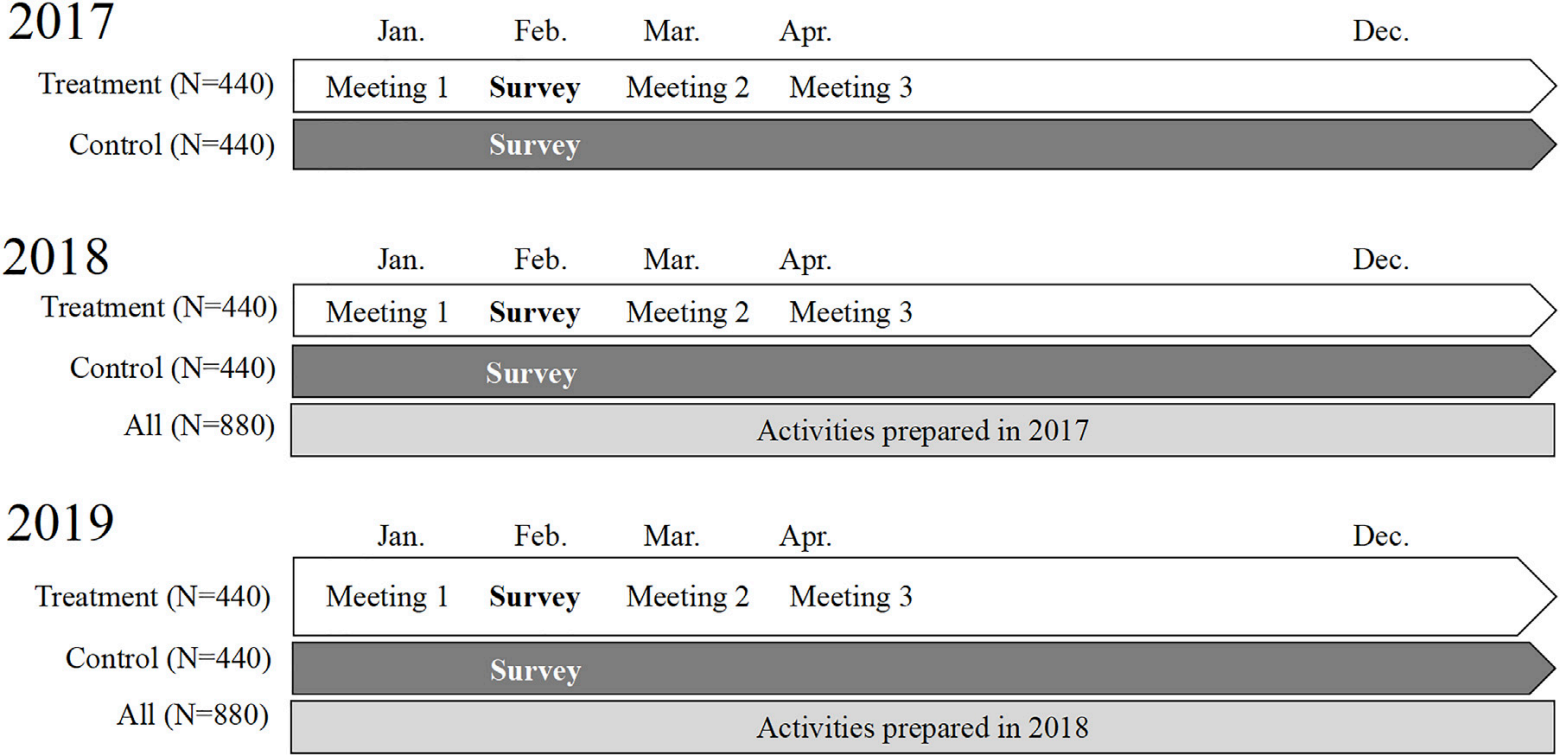
Intervention timeline (Can Intersectoral Interventions Reduce Substance Use in Adolescence? Evidence From a Multicentre Randomized Controlled Study, Foz do Iguaçu, 2022).^1^

As of 2018, the new activities were implemented in the institutions, available to all students (treatment and control). In sum, only the treatment group participated in the co-development of activities (three meetings) in all intervention years (2017–2019). In 2018–2019, both treatment and control groups had access to the activities developed. Figure 1 shows the intervention timeline with the meetings, survey and activities happening from 2017 to 2019. The main goal of this study is to measure the impact of students participating in activities developed by themselves, as opposed to participating in activities developed by their peers.

All students were informed and provided consent to participate in the study. Students in the control group were not harmed or excluded at any level during the course of this intervention.

Data collection occurred each year, managed by the project implementers through an electronic platform established for the purpose (http://caiunarede.pti.net.br).

### Econometric Estimation

The impact of the intervention was measured using a differences-in-differences (DiD) model. The DiD estimator provides an unbiased estimate of the treatment effect under the assumption that without the intervention, outcomes would have had the same evolution in both groups [18]. This assumption is reasonable thanks to the randomized controled study design.

We focus our analyses on three main outcomes that capture individual behavior targeted by the intervention: current tobacco, alcohol, and cannabis use. The survey collected self-reported consumption frequency over the last 30 days in seven categories: never, used in 1–2 days, 3–5 days, 6–9 days, 10–19 days, 20–29 days, everyday. We start by looking at whether adolescents consumed at all.

We estimate Probit regressions for each outcome variable as a function of the explanatory variables and the DiD terms, as specified in the following expression [19]:

$$\begin{array}{ll} & P\left(Y_{it}^{T,A,C}=1|Treat_{i},Time_{t},X_{it}\right)\\ & \;=\Phi \left(\alpha +\beta_{1}Treat_{i}+\beta_{2}Time_{t}+\beta_{3}Treat_{i}\times Time_{t}+\gamma X_{it}\right) \end{array}$$ (1)

In Equation 1, $Y_{it}^{T}$ is a binary variable indicating consumption of tobacco, $Y_{it}^{A}$ alcohol, and $Y_{it}^{C}$ cannabis in at least 1 out of the past 30 days. *Treat*_*i*_ identifies individuals in the treatment group. *Time*_*t*_ identifies the period after the intervention (2018 and 2019). The coefficient of main interest is *β*_3_, the coefficient on the interaction term. As the models are non-linear, the impact of the intervention on a specific outcome is obtained by calculating the average marginal effect that corresponds to *β*_3_. A series of individual characteristics (*X*_*it*_) are included in the model. These include age, gender, country, under and overweight Body-Mass Index (BMI) levels (below 15 and over 23 for girls, below 16 and over 22 for boys), an indicator of early sexual activity, and a peer effect. BMI was included as a proxy for health status; albeit not a perfect indicator, BMI is considered a valid and fairly objective indicator of individual health [20]. Early sexual activity is a proxy of personality (propensity for risky behaviour). Peer effect indicators are calculated using the leave-one-out mean strategy, which consists in the group average consumption excluding the individual (see Supplementary Files) [21]. Because consumption is a categorical variable, first we transformed it into an artificially continuous variable, by taking the mid-points of each category (e.g., category 3–5 days acquires the value 4). Lastly, Φ(.) denotes the cumulative distribution function of the standard normal distribution. All estimations cluster standard errors at the institutional level, to account for correlations between students from the same institution.

To investigate consumption along the intensive margin, we estimate additional probit models where we exclude those who never consumed in the past 30 days. In these models, the dependent variable takes the value 0 for those who consumed in 1–9 days, and the value 1 for those who consumed in at least 10 days. As a sensitivity check we also estimated ordered probit models, where the dependent variables are categorical instead of binary (see Supplementary Files).

## Results

### Descriptive Statistics

Table 1 shows the descriptive statistics for the different years and genders. The sample was balanced in terms of gender (434 boys and 446 girls). Around 42.7% (*n* = 376) of the participants were Brazilian, 33.2% (*n* = 292) Paraguayan, and 24.1% (*n* = 212) Argentinian. Nationality shares were quite stable over time; and so were average BMI and early sexual activity.

**TABLE 1:** Descriptive statistics (Can Intersectoral Interventions Reduce Substance Use in Adolescence? Evidence From a Multicentre Randomized Controlled Study, Foz do Iguaçu, 2022).

		2017		2018		2019	
		Boys	Girls	Boys	Girls	Boys	Girls
Age	Avg.	15.49	15.32	15.88	15.76	16.40	16.28
Brazil	Prop.	0.42	0.43	0.44	0.50	0.36	0.43
Paraguay	Prop.	0.31	0.35	0.33	0.34	0.35	0.39
Argentina	Prop.	0.27	0.21	0.23	0.17	0.30	0.17
BMI	Avg.	21.96	21.73	22.05	21.82	21.56	21.82
Early sexual activity	Prop.	0.15	0.13	0.15	0.14	0.12	0.16
Peer alcohol cons.	Avg. (days)	3	3	2	2	2	1
Peer cannabis cons.	Avg. (days)	2	2	1	1	1	1
Peer tobacco cons.	Avg. (days)	4	4	3	3	3	3
Total	N	446	434	318	327	223	235

**FIGURE 2:**
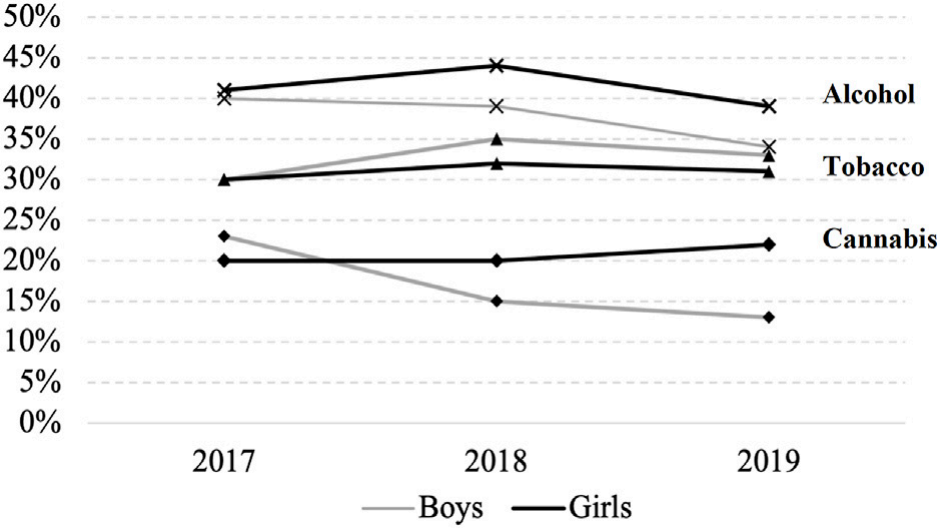
Frequency of any usage in the past 30 days, by gender over time (Can Intersectoral Interventions Reduce Substance Use in Adolescence? Evidence From a Multicentre Randomized Controlled Study, Foz do Iguaçu, 2022).

**FIGURE 3:**
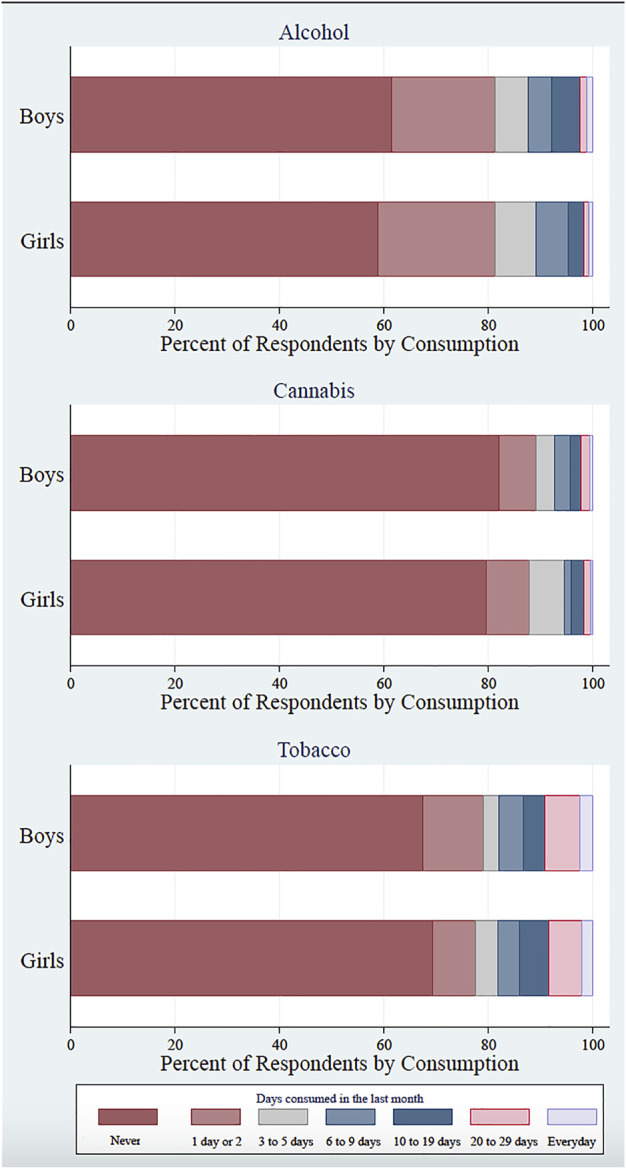
Percentage of respondents by level of frequency usage in the past 30 days, by gender and substance (Can Intersectoral Interventions Reduce Substance Use in Adolescence? Evidence From a Multicentre Randomized Controlled Study, Foz do Iguaçu, 2022).^2^

Figure 2 shows substance use (any consumption in the past 30 days) by gender and over time. Alcohol is the substance that more participants reported to consume at least once in the last month. The proportion of males consuming tobacco is larger, whereas cannabis consumption is more common among females in 2018 and 2019. Frequency of use by gender and substance is shown in Figure 3. Frequency of use is generally higher for tobacco. Female participants show less frequent consumption of alcohol and cannabis, but the percentage of students who didn’t consume any substance is higher among males. These values indicate that any substance use is more prevalent among girls, who consume with moderate frequency, while relatively fewer boys engage in any consumption, but those who do it with greater frequency.

### Main Results

The interaction coefficients in the DiD models are statistically significant and show negative impacts of the intervention on all indicators of interest: alcohol, cannabis and tobacco consumption in at least 1 day in the last month (Table 2). The likelihood of a teenager consuming alcohol in at least 1 day in the last month is 13 percentage points (pp) lower for those in the treatment group compared to the control group, after the intervention (p < 0.01). Further, the intervention reduced the likelihood of consuming either tobacco or cannabis in at least 1 day in the last month by 8 pp (p < 0.01). A one-day increase in the average frequency of consumption among peers increases the likelihood of consuming substances by 2 pp for alcohol, 4 pp for cannabis, and 3 pp for tobacco.

**TABLE 2:** Impacts of the intervention on any consumption in the last 30 days (marginal effects) (Can Intersectoral Interventions Reduce Substance Use in Adolescence? Evidence From a Multicentre Randomized Controlled Study, Foz do Iguaçu, 2022).

	Alcohol		Cannabis		Tobacco	
	(1)	(2)	(3)	(4)	(5)	(6)
Post-treat.	−0.01	0.03	−0.03**	0.03	0.03	0.07***
	(0.03)	(0.04)	(0.01)	(0.04)	(0.04)	(0.02)
Treatment	−0.01	−0.00	−0.02	−0.01	0.02	0.02
	(0.03)	(0.03)	(0.02)	(0.02)	(0.02)	(0.02)
**DiD**	−0.13***	−0.13***	−0.08***	−0.08***	−0.08***	−0.08***
	**(0.04)**	**(0.04)**	**(0.02)**	**(0.02)**	**(0.03)**	**(0.02)**
Girl	0.05	0.05	0.05	0.05	−0.02	−0.03
	(0.07)	(0.07)	(0.05)	(0.04)	(0.05)	(0.06)
Age	0.12***	0.09***	0.07***	0.03*	0.05**	0.03*
	(0.02)	(0.01)	(0.01)	(0.02)	(0.02)	(0.01)
Brazilian	−0.02	−0.00	−0.16**	−0.13	−0.02	0.02
	(0.05)	(0.06)	(0.08)	(0.08)	(0.05)	(0.05)
*BMI*_*under*_	0.02	0.04	0.04	0.05	−0.12	−0.09
	(0.14)	(0.14)	(0.08)	(0.08)	(0.10)	(0.10)
*BMI*_*over*_	0.01	0.01	0.00	0.00	−0.05**	−0.05**
	(0.04)	(0.04)	(0.03)	(0.04)	(0.02)	(0.03)
Early sex exposure	0.15*	0.15	0.08	0.07	0.15*	0.15*
	(0.09)	(0.09)	(0.06)	(0.05)	(0.08)	(0.08)
Peer avg. cons.		0.02**		0.04***		0.03***
		(0.01)		(0.01)		(0.01)

Note: Standard errors in parentheses are clustered at institution level.***p < 0.01, **p < 0.05, *p < 0.1.

Dependent variables are binary with the value 1 if the respondent consumed each substance at least once in the last month; and 0 otherwise. Peer is the (leave one out) average group consumption in days. BMI under and over are binary variables indicating whether each individual has an unhealthy BMI (by deficiency or excess) or not. Results showing the introduction of a peer effect in interaction with the intervention related variables–time, treatment and both (DiD)—had very similar results, presented in Supplementary File S3.

Regarding sociodemographic characteristics, Brazilian nationality is associated with a 16 pp (p < 0.05) lower likelihood of having consumed cannabis at least once in the last month, compared to Paraguayan and Argentinian nationalities, but the effect loses significance when the peer effect is included. Age is associated wit higher likelihood of consuming all substances: for example, one additional year of age increases the likelihood of any alcohol consumption in the past month by 9 pp (p < 0.01; model controlling for peer effects). Early initiation of sexual activity is associated with higher likelihood of consumption of alcohol and tobacco, but the impact is also reduced when the peer effect is included^2^.

In the second specification, we exclude participants that reported no consumption in the past 30 days and compare those who consumed in less than 10 days to those who consumed in at least 10 days (i.e., frequent or heavy consumption). Results in Table 3 show that the likelihood of frequent consumption was reduced by the intervention only in the case of cannabis, by 15 pp (p < 0.1).

**TABLE 3:** Impacts of the intervention on light vs. heavy consumption (marginal effects) (Can Intersectoral Interventions Reduce Substance Use in Adolescence? Evidence From a Multicentre Randomized Controlled Study, Foz do Iguaçu, 2022).

	Alcohol		Cannabis		Tobacco	
	(1)	(2)	(3)	(4)	(5)	(6)
Post-treat.	−0.16***	−0.06	−0.11**	0.00	−0.17***	−0.10*
	(0.03)	(0.03)	(0.05)	(0.05)	(0.05)	(0.05)
Treatment	−0.01	−0.02	−0.05	−0.03	−0.02	−0.01
	(0.03)	(0.03)	(0.05)	(0.05)	(0.05)	(0.05)
**DiD**	**0.01**	**0.02**	−0.15*	−0.16*	−**0.02**	−**0.04**
	**(0.05)**	**(0.05)**	**(0.09)**	**(0.09)**	**(0.07)**	**(0.07)**
Girls	−0.06**	−0.05**	−0.01	0.01	0.10***	0.08**
	(0.03)	(0.02)	(0.04)	(0.04)	(0.04)	(0.04)
Age	0.09***	0.02	0.10***	0.03	0.10***	0.06***
	(0.01)	(0.02)	(0.02)	(0.03)	(0.02)	(0.02)
Brazilian	−0.05*	0.03	0.03	0.09*	−0.33***	−0.24***
	(0.03)	(0.03)	(0.05)	(0.05)	(0.04)	(0.04)
*BMI*_*under*_	—	—	—	—	0.24	0.27*
					(0.15)	(0.15)
*BMI*_*over*_	−0.03	−0.01	−0.04	−0.03	−0.14***	−0.12***
	(0.03)	(0.02)	(0.04)	(0.04)	(0.04)	(0.04)
Early sex exposure	−0.04	−0.05	0.15***	0.12***	0.13***	0.11**
	(0.03)	(0.03)	(0.05)	(0.04)	(0.05)	(0.05)
Peer avg. cons.		0.04***		0.05***		0.03***
		(0.00)		(0.01)		(0.01)
Observations	773	773	371	371	627	627

Note: Standard errors in parentheses are clustered at institution level.***p < 0.01, **p < 0.05, *p < 0.1.

Dependent variables are binary with the value 1 if the respondent consumed each substance at least 10–19 days in the last month; and 0 otherwise. Peer is the (leave one out) average group consumption in days. BMI under and over are binary variables indicating whether each individual has an unhealthy BMI (by deficiency or excess) or not. Complete table in Supplementary File S3.

A one-day increase in the average frequency of consumption among peers increases the likelihood of consuming alcohol, cannabis and tobacco frequently by 4, 5 and 3 pp, respectively (p < 0.01). Interestingly, the inclusion of this variable in the models attenuates the effect associated with the “post” indicator, which otherwise suggested that participation in the activities, whether by treatment or control group adolescents, reduced the likelihood of frequent consumption among users. This implies a negative correlation between average frequency of consumption among the adolescents and participation in the activities. In other words, average frequency of consumption is lower in 2018–2019, the years when the activities are available.

As for the other variables, girls are less likely to be heavy alcohol users, but more likely to be heavy users of tobacco. Being 1 year older is associated with an increase in the likelihood of frequent consumption by 9–10 pp (p < 0.01). Early sexual activity is associated with consuming cannabis and tobacco frequently at the 1% significance level. Overweight teenagers (BMI above healthy level) are 12–14 pp less likely to be heavy smokers.

## Discussion

To summarize, results show that the intervention (i.e., involvement in the co-development of activities to tackle substance use) successfully reduced the use of alcohol, cannabis, and tobacco on the extensive margin (i.e., likelihood of use) and, to a lower extent, on the intensive margin (i.e., likelihood of frequent consumption among users). Compared to similar interventions, the magnitude of the impacts found here was larger [6]. In addition, users in the control group, who participated in the activities but not in their development, also decreased their consumption frequency. We find a positive relationship between substance consumption and risky behaviour, proxied by early sexual initiation. Consumption increases with age and average consumption frequency of peers. Because our consumption data refers to only 30 days of each intervention year, we are not equipped to infer about the harmfulness of the consumption behavior identified. We refrain from judging what is abusive consumption or “normal” consumption given the subjects’ age and cultural environment, and opt for reporting simply the effects on both the extensive and intensive margins.

Similar trends in frequency of alcohol consumption in adolescents, by sex and age, have been identified in other studies [22–25]. The increase in the consumption of alcohol and other substances among adolescents is still a controversy when it comes to the evidence on the effectiveness of interventions. For example, an intervention conducted in 2020 decreased the decision-making ability, leading to an increase in substance use in adolescents in the follow-up [26]. The increase in consumption of other substances was also observed after participation in school programs, multicomponent interventions, as well as interventions involving students, parents and teachers in other studies [27–29]. However, one particularity of our study is that the intervention was implemented by members of the institutions that have been working with the adolescents for a long time. We believe that the proximity between the project implementers and the participants contributed favourably to the success of the intervention in reducing substance use.

The association between substance use and migration and nationality has also been previously studied. A previous study in the region has highlighted the influence of interpersonal relationship patterns and cultural clashes on consumption [29]. The authors identified a reduction in substance use among adolescents in Latin America resulting from an intervention with parental content, that compared the parents’ culture of origin and consumption behaviour. In our case, Brazilian adolescents seem to have healthier habits, which can be related to having more stability compared to those with foreign nationality. This evidences the potential lack of integration of foreign students.

As for peer effects, our results show that group average consumption frequency influences individual consumption frequency, for all substances in almost all specifications. Our study also aligns with previous studies in what regards the relationship between risky behaviour and substance consumption [30]. That study showed the impact of a mobile app to change teenagers’ behaviour that helped reduce both substance consumption and risky sexual behaviour.

This work has several limitations. While we find robust results that intersectoral interventions can be a relevant tool to decrease substance consumption among adolescents, we do not have access to an extensive list of socioeconomic indicators, or consumption beyond the intervention window or beyond adolescence. Household composition, socioeconomic status, parental education and participation in the labour market may affect both adolescents’ consumption and the effectiveness of the intervention. We worked in partnership with institutions experienced in the subject of substance use, that had existing relationships with the adolescents. This likely affected the effectiveness of the intervention, implying it may not have replicable impacts in a setting where such straight relationships are not in place.

Overall, we argue that participatory intersectoral interventions, involving the subjects themselves, are effective to decrease substance use among teenagers, with benefits for both the adolescents involved in the development of the activities and those that only participate in them. Potentially, involvement of the subjects in co-development of activities raises awareness, engagement, attractiveness/adequacy of the activities, and integration of foreign and local students.

Recognizing the results of intersectoral initiatives to improve collaboration between adolescents and implementing agents is as important as monitoring and evaluating these programs for their effectiveness. Considering the positive results than have been recently found, this strategy can be a relevant tool for health policy decision makers to tackle substance use at an early stage, with support from different sectors, while avoiding the fragmentation of services and resources.

Based on ours and previous results, we consider this type of intervention to be very effective in engaging participants and reducing harmful behavior, which may subsequently improve their health outcomes, general well-being, and socioeconomic conditions. We believe governments and institutions should continue to and increase financing of intersectoral interventions at an early stage, while also developing research studies that identify the most effective mechanisms for different individuals and adapt the interventions to their needs.

## Footnotes

1 Note: Meeting 1- Health education training (Health educ); 2- Survey indicators analysis (Indic.): 3- Development of proposals (Prop.); N participants=880; Meetings are conducted by project implementers.

2
Figure 3 shows the percentage of participants per reported frequency of use, in days out of the last 30 days, by gender and substance.
