# Supplementary material for: Can Intersectoral Interventions Reduce Substance Use in Adolescence? Evidence From a Multicentre Randomized Controlled Study
Source: Int J Public Health. 2022 Aug 26;67:1604677. doi: 10.3389/ijph.2022.1604677 (PMC9458850; doi:10.3389/ijph.2022.1604677)
Supplement: Supplementary file 1 [file DataSheet1.pdf]

Supplementary material

S1 Supplementary file 1 — Figures

Figure S1: Figure A1. Region map

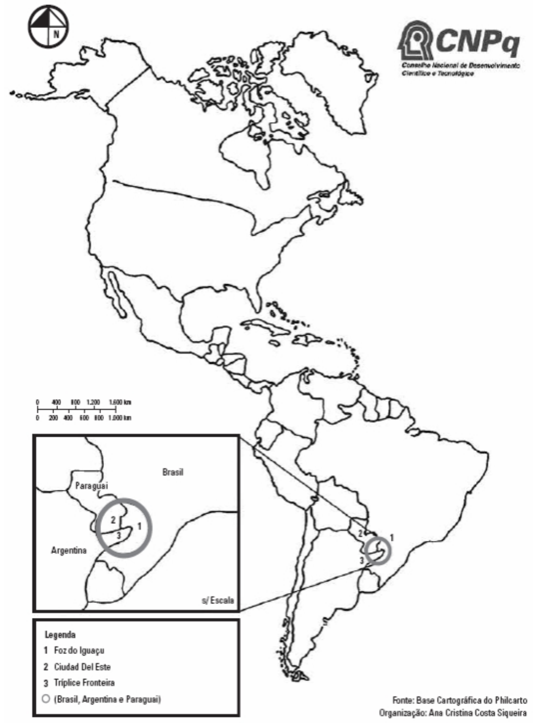

Figure S2: Average frequency of consumption by gender, substance and year

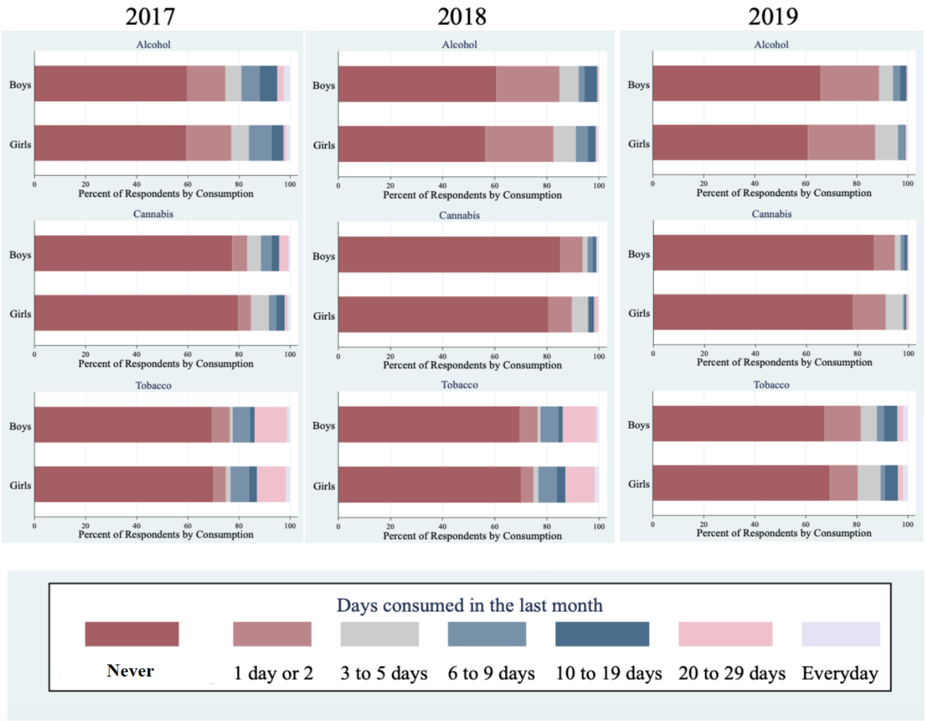

S2 Supplementary file 2 — Additional information

Table S1: Human Development Index by intervention region

Region	Population	HDI
Iguazu River Mouth– Parana, Brazil	264,044 inhab.	0.751
Coronel Oviedo – Caaguazu, Paraguay	117,514 inhab.	0.521
Puerto Iguazu – Misiones, Argentina	80,020 inhab.	0.817

Table S2: Number of adolescents by institution

Inst. Code	Action area	Country	Total enrolled	Participants
1	Social services	Brazil	818	84
2	Social services	Brazil	38	4
3	Social services	Brazil	140	14
4	Social services	Brazil	10	1
5	Social services	Brazil	38	4
6	Education	Brazil	400	41
7	Education	Brazil	327	34
8	Education	Brazil	403	41
9	Education	Brazil	200	21
10	Education	Brazil	291	27
11	Education	Brazil	187	19
12	Education	Brazil	66	7
13	Justice	Brazil	59	6
14	Education	Brazil	326	33
15	Sports	Brazil	40	4
16	Health	Brazil	351	36
17	Health	Brazil	20	9
18	Education	Brazil	320	144
19	Social services	Argentina	20	9
20	Social services	Argentina	20	9
	Justice	Argentina	90	41
	Health	Argentina	170	42
	Justice	Argentina	186	46
21	Social services	Paraguay	100	25
22	Sports	Paraguay	610	150
23	Education	Paraguay	120	29
Total			5350	880

S3 Supplementary file 3 — Complete tables

Table S3: Alcohol, tobacco and consumption by gender and year

	0 consump.		1 - 9 days consump.		10 - 30 days consump.	
	(1)	(2)	(3)	(4)	(5)	(6)
**Tobacco**						
Post-treat.	0.00	-0.09*	-0.00	0.04*	-0.00	0.06*
	(0.04)	(0.05)	(0.01)	(0.02)	(0.02)	(0.03)
Treatment	-0.01	-0.01	0.00	0.01	0.01	0.01
	(0.02)	(0.03)	(0.01)	(0.01)	(0.01)	(0.02)
DiD	0.08***	0.17	-0.03***	-0.06*	-0.05***	-0.10
	(0.02)	(0.10)	(0.01)	(0.04)	(0.02)	(0.07)
Peer		-0.03***		0.01***		0.02***
		(0.01)		(0.00)		(0.01)
Post-treat x Peer		0.01		-0.01		-0.01
		(0.02)		(0.01)		(0.01)
Treatment x Peer		-0.00		0.00		0.00
		(0.00)		(0.00)		(0.00)
DiD x Peer		-0.03		0.01		0.02
		(0.03)		(0.01)		(0.02)
**Alcohol**						
	0 consump.		1 - 9 days consump.		10 - 30 days consump.	
	(1)	(2)	(3)	(4)	(5)	(6)
Post-treat.	0.06***	0.04	-0.04***	-0.03	-0.02***	-0.01
	(0.02)	(0.06)	(0.02)	(0.04)	(0.01)	(0.02)
Treatment	0.01	0.03	-0.01	-0.02	-0.00	-0.01
	(0.03)	(0.03)	(0.02)	(0.02)	(0.01)	(0.01)
DiD	0.11***	0.10*	-0.07***	-0.07*	-0.03**	-0.03*
	(0.03)	(0.05)	(0.02)	(0.04)	(0.01)	(0.02)
Peer		-0.03***		0.02***		0.01**
		(0.01)		(0.00)		(0.00)
Post-treat x Peer		-0.02		0.01		0.01
		(0.01)		(0.01)		(0.00)
Treatment x Peer		-0.01		0.00		0.00
		(0.01)		(0.00)		(0.00)
DiD x Peer		-0.00		0.00		0.00
		(0.02)		(0.01)		(0.00)
**Cannabis**						
	0 consump.		1 - 9 days consump.		10 - 30 days consump.	
	(1)	(2)	(3)	(4)	(5)	(6)
Post-treat.	0.05***	-0.01	-0.03***	0.01	-0.02***	0.00
	(0.01)	(0.02)	(0.01)	(0.02)	(0.00)	(0.01)
Treatment	0.03	-0.00	-0.02	0.00	-0.01	0.00
	(0.02)	(0.03)	(0.02)	(0.02)	(0.01)	(0.01)
DiD	0.08***	0.12***	-0.06***	-0.08***	-0.03***	-0.04***
	(0.02)	(0.02)	(0.01)	(0.02)	(0.01)	(0.01)
Peer		-0.04***		0.03***		0.01***
		(0.01)		(0.00)		(0.00)
Post-treat x Peer		-0.01		0.01		0.00
		(0.01)		(0.01)		(0.00)
Treatment x Peer		0.01		-0.01		-0.00
		(0.01)		(0.01)		(0.00)
DiD x Peer		-0.02		0.02		0.01
		(0.02)		(0.01)		(0.01)
Observations	1.982	1.982	1.982	1.982	1.982	1.982

Note: Standard errors in parentheses.*** $p<0.01$, ** $p<0.05$, * $p<0.1$

Table S4: Probit estimation – light vs. heavy consumers

	Tobacco		Cannabis		Alcohol	
	(1)	(2)	(3)	(4)	(5)	(6)
Post-treat.	-0.17***	-0.18*	-0.16***	-0.15***	-0.11**	-0.00
	(0.05)	(0.11)	(0.03)	(0.05)	(0.05)	(0.10)
Treatment	-0.02	0.04	-0.01	-0.03	-0.05	0.02
	(0.05)	(0.10)	(0.03)	(0.05)	(0.05)	(0.11)
DiD	-0.02	-0.16	0.01	0.07	-0.15*	-0.18
	(0.07)	(0.14)	(0.05)	(0.08)	(0.09)	(0.15)
Peer		0.02*		0.03***		0.06***
		(0.01)		(0.01)		(0.02)
Post-treat x Peer		0.02		0.03**		0.01
		(0.02)		(0.01)		(0.03)
Treatment x Peer		-0.01		0.00		-0.02
		(0.02)		(0.01)		(0.02)
DiD x Peer		0.03		-0.02		-0.00
		(0.03)		(0.02)		(0.04)
Woman	0.10***	0.08**	-0.06**	-0.05**	-0.01	0.01
	(0.04)	(0.04)	(0.03)	(0.02)	(0.04)	(0.04)
Age	0.10***	0.07***	0.09***	0.03*	0.10***	0.03
	(0.02)	(0.02)	(0.01)	(0.02)	(0.02)	(0.03)
Brazilian	-0.33***	-0.25***	-0.05*	0.02	0.03	0.08*
	(0.04)	(0.04)	(0.03)	(0.03)	(0.05)	(0.05)
BMI_{under}	0.24	0.27*	-	-	-	-
	(0.15)	(0.15)				
BMI_{over}	-0.14***	-0.12***	-0.03	-0.01	-0.04	-0.03
	(0.04)	(0.04)	(0.03)	(0.02)	(0.04)	(0.04)
Early sex exposure	0.13***	0.12**	-0.04	-0.04	0.15***	0.12***
	(0.05)	(0.05)	(0.03)	(0.03)	(0.05)	(0.05)
Observations	627	627	773	773	371	371

Robust standard errors in parentheses; *** p<0.01, ** p<0.05, * p<0.1

Table S5: Ordered Probit results for tobacco consumption

**Tobacco**	0 consumption		1 to 9 days		10 days or more	
	(1)	(2)	(3)	(4)	(5)	(6)
Post-treat.	0.00	-0.09*	-0.00	0.04*	-0.00	0.06*
	(0.04)	(0.05)	(0.01)	(0.02)	(0.02)	(0.03)
Treatment	-0.01	-0.01	0.00	0.01	0.01	0.01
	(0.02)	(0.03)	(0.01)	(0.01)	(0.01)	(0.02)
DiD	0.08***	0.17	-0.03***	-0.06*	-0.05***	-0.10
	(0.02)	(0.10)	(0.01)	(0.04)	(0.02)	(0.07)
Peer		-0.03***		0.01***		0.02***
		(0.01)		(0.00)		(0.01)
Post-treat x Peer		0.01		-0.01		-0.01
		(0.02)		(0.01)		(0.01)
Treatment x Peer		-0.00		0.00		0.00
		(0.00)		(0.00)		(0.00)
DiD x Peer		-0.03		0.01		0.02
		(0.03)		(0.01)		(0.02)
Woman	0.01	0.02	-0.00	-0.01	-0.01	-0.01
	(0.05)	(0.06)	(0.02)	(0.02)	(0.03)	(0.04)
Age	-0.07***	-0.03**	0.02**	0.01**	0.04***	0.02**
	(0.02)	(0.01)	(0.01)	(0.01)	(0.01)	(0.01)
Brazilian	0.07	0.01	-0.03	-0.01	-0.04	-0.01
	(0.05)	(0.05)	(0.02)	(0.02)	(0.03)	(0.03)
BMI_{under}	0.09	0.05	-0.03	-0.02	-0.05	-0.03
	(0.11)	(0.10)	(0.04)	(0.04)	(0.07)	(0.06)
BMI_{over}	0.07***	0.06***	-0.03***	-0.02***	-0.04***	-0.04***
	(0.02)	(0.02)	(0.01)	(0.01)	(0.01)	(0.01)
Early sex exposure	-0.14*	-0.14*	0.05*	0.06*	0.09**	0.09**
	(0.08)	(0.07)	(0.03)	(0.03)	(0.04)	(0.04)
Observations	1.983	1.983	1.983	1.983	1.983	1.983

Robust standard errors in parentheses; *** p<0.01, ** p<0.05, * p<0.1
Dependent variable is an ordered variable indicating the frequency of consumption in the last 30 days - with the value 0 for 0 days; value 1 if consumed between 1 and 9 days; value 2 if consumed more than 10 days. DiD is the difference-in-differences coefficient. Peer is the (leave one out) average group consumption in days. BMI under and over are binary variables indicating whether each individual has an unhealthy BMI (by deficiency or excess) or not.

Table S6: Ordered Probit results for cannabis consumption

**Drugs**	0 consumption		1 to 9 days consumption		10 days or more	
	(1)	(2)	(3)	(4)	(5)	(6)
Post-treat.	0.05***	-0.01	-0.03***	0.01	-0.02***	0.00
	(0.01)	(0.02)	(0.01)	(0.02)	(0.00)	(0.01)
Treatment	0.03	-0.00	-0.02	0.00	-0.01	0.00
	(0.02)	(0.03)	(0.02)	(0.02)	(0.01)	(0.01)
DiD	0.08***	0.12***	-0.06***	-0.08***	-0.03***	-0.04***
	(0.02)	(0.02)	(0.01)	(0.02)	(0.01)	(0.01)
Peer		-0.04***		0.03***		0.01***
		(0.01)		(0.00)		(0.00)
Post-treat x Peer		-0.01		0.01		0.00
		(0.01)		(0.01)		(0.00)
Treatment x Peer		0.01		-0.01		-0.00
		(0.01)		(0.01)		(0.00)
DiD x Peer		-0.02		0.02		0.01
		(0.02)		(0.01)		(0.01)
Woman	-0.04	-0.04	0.03	0.03	0.01	0.01
	(0.04)	(0.04)	(0.03)	(0.03)	(0.01)	(0.01)
Age	-0.08***	-0.04**	0.05***	0.02**	0.03***	0.01***
	(0.01)	(0.01)	(0.01)	(0.01)	(0.00)	(0.00)
Brazilian	0.16**	0.12*	-0.11**	-0.08*	-0.05**	-0.04*
	(0.07)	(0.07)	(0.05)	(0.05)	(0.02)	(0.02)
BMI_{under}	-0.01	-0.03	0.01	0.02	0.00	0.01
	(0.06)	(0.06)	(0.04)	(0.04)	(0.02)	(0.02)
BMI_{over}	0.00	0.01	-0.00	-0.00	-0.00	-0.00
	(0.03)	(0.03)	(0.02)	(0.02)	(0.01)	(0.01)
Early sex exposure	-0.09*	-0.09**	0.06*	0.06**	0.03*	0.03**
	(0.05)	(0.04)	(0.03)	(0.03)	(0.02)	(0.01)
Observations	1.982	1.982	1.982	1.982	1.982	1.982

Robust standard errors in parentheses; *** p<0.01, ** p<0.05, * p<0.1
Dependent variable is an ordered variable indicating the frequency of consumption in the last 30 days - with the value 0 for 0 days; value 1 if consumed between 1 and 9 days; value 2 if consumed more than 10 days. DiD is the difference-in-differences coefficient. Peer is the (leave one out) average group consumption in days. BMI under and over are binary variables indicating whether each individual has an unhealthy BMI (by deficiency or excess) or not.

Table S7: Ordered Probit results for alcohol consumption

**Alcohol**	0 consumption		1 to 9 days consumption		10 days or more	
	(1)	(2)	(3)	(4)	(5)	(6)
Post-treat.	0.06***	0.04	-0.04***	-0.03	-0.02***	-0.01
	(0.02)	(0.06)	(0.02)	(0.04)	(0.01)	(0.02)
Treatment	0.01	0.03	-0.01	-0.02	-0.00	-0.01
	(0.03)	(0.03)	(0.02)	(0.02)	(0.01)	(0.01)
DiD	0.11***	0.10*	-0.07***	-0.07*	-0.03**	-0.03*
	(0.03)	(0.05)	(0.02)	(0.04)	(0.01)	(0.02)
Peer		-0.03***		0.02***		0.01**
		(0.01)		(0.00)		(0.00)
Post-treat x Peer		-0.02		0.01		0.01
		(0.01)		(0.01)		(0.00)
Treatment x Peer		-0.01		0.00		0.00
		(0.01)		(0.00)		(0.00)
DiD x Peer		-0.00		0.00		0.00
		(0.02)		(0.01)		(0.00)
Woman	-0.02	-0.03	0.02	0.02	0.01	0.01
	(0.07)	(0.07)	(0.05)	(0.05)	(0.02)	(0.02)
Age	-0.13***	-0.09***	0.09***	0.06***	0.04***	0.03***
	(0.01)	(0.01)	(0.01)	(0.01)	(0.01)	(0.00)
Brazilian	0.04	0.01	-0.03	-0.01	-0.01	-0.00
	(0.04)	(0.05)	(0.03)	(0.03)	(0.01)	(0.01)
BMI_{under}	0.03	0.00	-0.02	-0.00	-0.01	-0.00
	(0.11)	(0.11)	(0.07)	(0.07)	(0.04)	(0.03)
BMI_{over}	0.00	-0.01	-0.00	0.00	-0.00	0.00
	(0.03)	(0.03)	(0.02)	(0.02)	(0.01)	(0.01)
Early sex exposure	-0.12*	-0.11	0.08	0.08	0.04**	0.04*
	(0.07)	(0.07)	(0.05)	(0.05)	(0.02)	(0.02)
Observations	1.983	1.983	1.983	1.983	1.983	1.983

Robust standard errors in parentheses; *** p<0.01, ** p<0.05, * p<0.1
Dependent variable is an ordered variable indicating the frequency of consumption in the last 30 days - with the value 0 for 0 days; value 1 if consumed between 1 and 9 days; value 2 if consumed more than 10 days. DiD is the difference-in-differences coefficient. Peer is the (leave one out) average group consumption in days. BMI under and over are binary variables indicating whether each individual has an unhealthy BMI (by deficiency or excess) or not.
